# Novel Insights into the Therapeutic Potential of Lung-Targeted Gene Transfer in the Most Common Respiratory Diseases

**DOI:** 10.3390/cells11060984

**Published:** 2022-03-12

**Authors:** Malik Bisserier, Xiao-Qing Sun, Shahood Fazal, Irene C. Turnbull, Sébastien Bonnet, Lahouaria Hadri

**Affiliations:** 1 Cardiovascular Research Institute, Icahn School of Medicine at Mount Sinai, 1470 Madison Avenue, New York, NY 10029, USA; malik.bisserier@mssm.edu (M.B.); shahood.fazal@icahn.mssm.edu (S.F.); irene.turnbull@mssm.edu (I.C.T.); 2 Department of Pulmonary Medicine, Amsterdam Cardiovascular Sciences, Amsterdam UMC, Vrije Universiteit Amsterdam, 1081 HV Amsterdam, The Netherlands; xiqi.sun@outlook.com; 3 Pulmonary Hypertension Research Group, Québec Heart and Lung Institute Research Centre, Québec, QC G1V4G5, Canada; sebastien.bonnet@criucpq.ulaval.ca; 4 Department of Medicine, Laval University, Québec, QC G1V4G5, Canada

**Keywords:** gene therapy, treatment, respiratory disease, AAV, gene editing, nanoparticles, pulmonary hypertension, COPD, lung fibrosis, COVID-19

## Abstract

Over the past decades, a better understanding of the genetic and molecular alterations underlying several respiratory diseases has encouraged the development of new therapeutic strategies. Gene therapy offers new therapeutic alternatives for inherited and acquired diseases by delivering exogenous genetic materials into cells or tissues to restore physiological protein expression and/or activity. In this review, we review (1) different types of viral and non-viral vectors as well as gene-editing techniques; and (2) the application of gene therapy for the treatment of respiratory diseases and disorders, including pulmonary arterial hypertension, idiopathic pulmonary fibrosis, cystic fibrosis, asthma, alpha-1 antitrypsin deficiency, chronic obstructive pulmonary disease, non-small-cell lung cancer, and COVID-19. Further, we also provide specific examples of lung-targeted therapies and discuss the major limitations of gene therapy.

## 1. Introduction

Chronic respiratory diseases are one of the main leading causes of death and disability worldwide [1]. Many respiratory conditions are currently without curative treatment, and there are only limited treatment options. Given its accessibility and the identification of genetic alterations in many respiratory diseases, the lung is an attractive organ for targeted treatment delivery, especially for gene therapy. Gene therapy opens new avenues for treating inherited and acquired diseases by delivering exogenous genetic materials into cells or tissues to restore physiological protein expression and/or activity. Genetic materials include plasmid DNA, antisense oligonucleotides, mRNA, and peptide nucleic acids. To date, 23 gene therapy drugs have been approved for clinical application, and hundreds of clinical trials are underway to explore further possibilities [2]. In recent years, a better understanding of the genetic and molecular mechanisms of respiratory diseases has encouraged the application of gene therapy in this field.

Since Theodore Friedmann introduced gene therapy for monogenic diseases in 1972, the techniques of gene editing, as well as methods of gene delivery, have significantly advanced over the past decades [3]. The recent development of gene editing, especially the technique involving the clustered regularly interspaced short palindromic repeats (CRISPR)-associated system (Cas), has transformed the field and led to an exponential growth of gene therapy studies and clinical trials since 2012. Meanwhile, local delivery methods for gene therapy have faced challenges in reaching the target, especially for respiratory diseases, due to the structural and functional complexities of the lung and a unique set of immune cells. Therefore, multiple vectors have been developed and investigated to deliver cargoes for gene addition and gene-editing components, with the ultimate goal of overcoming the physical and biological barriers imposed by the lung anatomy and innate immune defenses.

**Figure 1:**
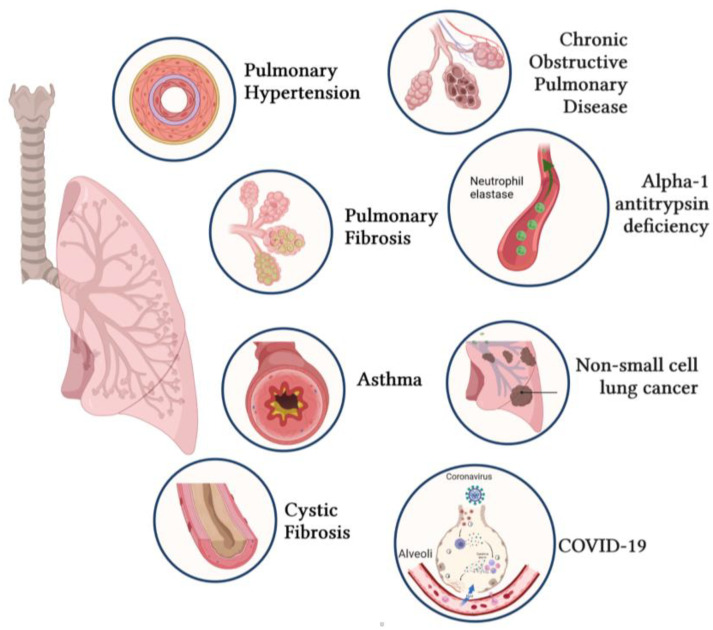
Common respiratory diseases associated with genetic disorders or molecular alterations. Over the past decades, progress in molecular profiling has provided critical insights into the genetic profile of patients with respiratory diseases, such as pulmonary arterial hypertension, pulmonary fibrosis, asthma, cystic fibrosis, chronic obstructive pulmonary disease, alpha-1 antitrypsin deficiency, non-small-cell lung cancer, and COVID-19. Here, we display schematic representations of the main pathological features of the diseases mentioned above, including vascular remodeling, mucus gland hyperplasia, inflammatory cell infiltrates, bronchial smooth muscle hypertrophy, fibrosis, emphysema, and tumor formation. Created with BioRender.com (accessed on 3 March 2022).

With the progress in genetic approaches, a wide range of defective gene expression profiles have been identified, which are highly related to the pathogenesis of multiple respiratory diseases, such as bone morphogenetic protein type 2 receptor (*BMPR2*) in pulmonary arterial hypertension (PAH) and cystic fibrosis transmembrane conductance regulator *(CFTR)* in cystic fibrosis (CF) [4,5]. This has prompted gene therapy to be considered as a new encouraging therapeutical approach for respiratory diseases. Over the past decades, lung-targeted gene therapy has improved, with better vector design, enhanced delivery, and higher efficacy [6]. The therapeutic potential of gene therapy has been further investigated in various respiratory diseases (Figure 1), including PAH, idiopathic pulmonary fibrosis (IPF), asthma, CF, chronic obstructive pulmonary disease (COPD), alpha-1 antitrypsin (AAT) deficiency, lung cancer, and coronavirus disease 2019 (COVID-19).

In this review, we will review (1) different types of viral and non-viral vectors as well as gene-editing techniques that are currently being used in respiratory diseases; and (2) examples of lung-targeted gene therapy for the treatment of respiratory diseases. Further, we also discuss the limitations of gene therapy in preclinical and human studies.

## 2. Vectors of Gene Therapy in Respiratory Diseases

### 2.1. Viral Vectors

#### 2.1.1. Retroviral and Lentiviral Vectors

Retrovirus and lentivirus genomes are characterized by two long terminal repeats at either end of the genome and a packaging sequence. A transgene cassette up to 8 kb can be inserted in the space by removing native genes, such as *GAG*, *POL*, and *ENV*. The RNA genome is converted into double-stranded DNA that can randomly integrate into the host’s chromosomal DNA during the viral life cycle. As a result, retroviral and lentiviral vectors have been investigated in proliferating cells, with attempts to achieve stable transduction by transmitting the therapeutic gene to daughter cells [7]. Consequently, the use of retroviral vectors for in vivo applications is not recommended, as lung cells only show low cell proliferation rates [8].

Lentiviral vectors can infect post-mitotic cells [9] with higher transduction rates. Lentiviral vectors have been broadly used in clinical trials for treating Parkinson’s disease and immunodeficiency disorders [10]. Moreover, lentiviral vectors have been approved for treating certain lymphomas and leukemias, which mainly involve the ex vivo treatment of hematopoietic stem cells [11]. Recently, several preclinical studies have shown encouraging data with lentiviral vectors regarding safety [12,13]; further clinical studies are needed to evaluate the long-term safety and efficacy. The use of lentiviral vectors for lung gene delivery is limited due to the barriers in the lung and the low localized expression of cellular receptors on the apical surface of the lung epithelial cells [14]. This can be overcome, in part, by using pseudotyped lentiviral vectors from diverse origins, such as Ebola, Sendai virus, or influenza. Recent studies have found multiple pseudotyped lentiviral vectors that can achieve robust apical transduction in epithelial cells in vivo, such as G64-pseudotyped or Sendai virus-pseudotyped lentiviral vectors [13,15,16,17]. However, the potential integration of the transgene into the host genome limits their use in clinical settings. The risk of random integration could also potentially activate proto-oncogenes or inactivate tumor suppressor genes [18].

#### 2.1.2. Adenovirus Vectors

Recombinant adenovirus (Ad) vectors are characterized by a natural tropism for airway epithelial cells in the respiratory tract, making them efficient vehicles for lung gene transfer. Ad vectors consist of a double-stranded genome. E1 gene deletion allows us to prevent vector replication and provides greater packaging space for the transgene [19]. These vectors can transduce proliferating and non-dividing cells. They are relatively easy to produce and purify at a large scale. Due to the highly immunogenic nature of Ad vectors, host immunity may limit the gene expression to 2–3 weeks [20]. For example, Ad-mediated gene transfer can cause an immune response and apoptosis in endothelial cells in organ-cultured pulmonary arteries, which leads to low levels of gene transfer [21]. The genetic material of Ad vectors is not integrated into the host’s cells genome, but rather remains episomal in the nucleus. Thus, the long-term episomal persistence of transgenes depends on the proliferative state of target cells. However, proliferating cells dilute the transgene expression, since only daughter cells express the exogenous transgene. As previously mentioned, Ad vectors induce humoral and cellular immune responses [22]. They are, therefore, unsuitable for repeated administration. Similar to retroviral and lentiviral vectors, the receptors for entry and internalization are mainly located in the basolateral surface. Since epithelial cells have tight junctions, vector–receptor interactions may be limited. Despite these limitations, there is an extensive safety record for lung gene therapy [8]. Second-generation Ad vectors have been developed to minimize immunogenicity and reduce the production of viral antigens by deleting the E2 and E4 viral genes [20]. These new vectors also partially reduce the vector-induced inflammatory response. More recently, “gutted/helper-dependent” adenovirus (HD-Ad) vectors have been developed by removing viral genes, with a reduced immunogenicity of Ad vectors and a large packaging capacity of up to 36 kb [23]. HD-Ad vectors expressing *CFTR* can efficiently transduce and restore CFTR activity in vitro and in vivo [24].

#### 2.1.3. Adeno-Associated Viruses (AAV) and Their Vectors

AAV are nonpathogenic, single-stranded parvoviruses and do not cause any diseases. The *Rep* and *Cap* genes are bound by two inverted terminal repeats in wild-type AAV. In recombinant AAV (rAAV) vectors, the *Rep* and *Cap* genes are replaced by the cDNA encoding the transgene of interest. Recombinant AAV vectors are not integrated into the host genome. The production of AAV requires the co-transfection of the AAV construct with a plasmid containing a helper. AAV can also be produced by infecting packaging cell lines with a recombinant helper containing the rAAV genome. AAV vectors have demonstrated better transduction efficiency and long-term transgene expression in quiescent and dividing cells.

Contrary to Ad vectors, AAV vectors do not induce a strong immune response. AAV have a broad host range and have been commonly used to transfer genes to the airway epithelium, alveolar epithelium, pulmonary vascular endothelium, and pleural mesothelioma [25]. Initially, only two serotypes of the AAV vectors, 2 and 5, were available. More than 100 serotypes have since been developed to enhance cellular and tissue tropism, expand their therapeutic applications to several diseases, and limit off-target effects. However, the size of the expression cassette is limited at 5 kb, which may narrow their application to “small” transgenes. Recently, the application of a smaller Cas variant or dual AAV vectors may provide a solution to the packaging size limitation. Indeed, the strategy of dual AAV vectors has been shown to reach effective transduction levels in pig and mice lungs [26,27]. The packaging capacity of AAV vectors can be increased by creating a parvovirus chimera, with the rAAV genome packaged in the capsid of another parvovirus, such as human bocavirus (HBoV) or gorilla bocavirus (GBoV). Recent studies have confirmed the transduction efficacy of rAAV/HBoV and rAAV/GBoV in primary human airway epithelial cells, lung organoids, and ferret lungs [28,29,30]. Currently, the use of AAV vectors is among the most frequently used strategies for gene therapy. While they have shown promising results in patients, one major concern is that much of the human population has already been exposed to various AAV serotypes, and as a result, they may already exhibit neutralizing antibodies that can significantly impair future gene transfer applications [31]. New recombinant AAV (rAAV), such as site-mutated AAV, are now being developed and optimized to evade the immune response associated with pre-existing neutralizing antibodies, while improving the AAV tropism and increasing transduction efficiency [32,33,34]. For example, site-directed mutagenesis (tyrosine to phenylalanine) has been used to impair the ubiquitination of surface-exposed tyrosine residues, which significantly decreased proteasome-mediated degradation and improved vector transduction [32,35]. Previous studies from Petrs-Silva et al. reported that the delivery of mutated AAV2, AAV8, and AAV9 in mouse retina enhanced transduction efficiency using mutated AAV2, AAV8, and AAV9 [36]. Similarly, Martini et al. investigated whether tyrosine mutations improve the gene transfer efficiency of AAV8 in the lung after intratracheal delivery. Consistently, they found that tyrosine-mutant AAV8 vectors enhanced the transduction efficiency in the lung without inducing any significant changes in lung mechanics, morphometry, or inflammatory response [37]. Another study assessed the effects of single-strand or self-complementary recombinants of AAV vectors containing single or multiple tyrosine-to-phenylalanine (Y-F) mutations in capsid surface-exposed residues on AAV serotypes 2, 8, or 9 [38]. The authors identified six rAAV vectors that showed higher transduction efficacy in CF bronchial epithelial cells by overcoming the intracellular trafficking and second-strand DNA synthesis limitations [38].

#### 2.1.4. Other Viral Vectors

Many other viruses, such as polyomaviruses, Vaccinia virus, baculovirus, and Sendai virus, have been used for lung gene therapy. Polyomaviruses and John Cunningham virus can provide sustained transgene expression by integrating into the host genome in dividing and non-dividing cells. These vectors can be produced and purified in high titers; however, they showed a limited packaging capacity of approximately 2.5–5 kb and potential for random integration [39]. Vaccinia virus (VV) vectors allow the insertion of large DNA fragments up to 25 kb in size. They represent a promising tool for lung gene transfer as they can infect most cells. Conversely, VV vectors may induce cytopathic effects and immune responses. They may also have limited efficacy due to neutralizing antibodies associated with infections. These properties have been further investigated for treating lung cancer [40]. Baculovirus expression vectors (BEV) have been used for recombinant protein expression and can carry up to 38 kb. BEV cannot replicate in mammalian cells and have no pre-existing BEV immunity in humans. The short-term transgene expression, the entry process through the basolateral membrane, and rapid virus inactivation by serum complements represent major challenges that should be considered for vector optimization [41]. Finally, Sendai virus (SeV)-derived vectors have shown a transduction efficiency in mice in vivo that is 3–4 logs higher than Ad5 or plasmid/liposomes [42]. Since transgene expression is transient, repeated administration leads to diminished gene expression. Pseudotyping SeV envelope proteins onto lentivirus can overcome this limitation [13].

### 2.2. Non-Viral Vectors

Different types of non-viral vectors can be used to provide the therapeutic genetic material as naked DNA or RNA (messenger RNA (mRNA), short double-stranded RNA, including small interfering RNA (iRNA) and microRNA (miRNA) mimics, or modified mRNA (modRNA)) or complexed with other macromolecules to enhance the efficiency of cell entry. RNA-based gene therapy has successfully overcome the limitations previously identified with viral vectors such as adenovirus, AAV, retroviral, and lentiviral vectors, including (1) the size capacity of the expression cassette, (2) immunogenicity, and (3) insertional mutagenesis. The advantages of non-viral systems include the ease of vector production, greater expression cassette size, and relatively minimal biosafety risks. Importantly, RNA does not require nuclear localization or transcription, whereas the transfer of DNA cargo requires translocation into the nucleus. An additional benefit is the negligible risk of genomic integration of the delivered sequence. Even though these vectors have significant advantages, they only displayed short-term expression, limited tropism, and moderate efficiency in vivo [43].

#### Nanoparticle-Based Therapeutics

Recent advances in nanoparticle-based therapeutics have led to new delivery systems that can deliver siRNAs, exogenous DNA, and mRNA to cells. Liposomes combined with plasmids have been used to enhance the internalization of the genetic material into the cytosol via endocytosis. Nanoparticles consist of a nucleic acid complex with other materials, such as lipid, polymers, peptides, and polysaccharides [44]. Solid lipid nanoparticles that can remain solid at physiological temperatures have been employed because they stay protected from nucleic acid degradation caused by the nuclease. Despite the risk of cytotoxicity, synthetic polymers, such as polyethyleneimine (PEI) or polyethylene glycol (PEG), are commonly used for gene transfer, given their higher efficiency. Another approach relies on molecular conjugates, consisting of a polylysine-DNA complex and a macromolecule receptor ligand that can be internalized by the target cells [45]. Lipid nanoparticles (LNPs) are formulated by combining lipid-based components and siRNA or modRNA; the combination of the organic and aqueous components induces hydrophobic/hydrophilic interactions, resulting in the formation of nanoparticles [46]. The lipidoid components can be optimized through combinatorial chemistry approaches to achieve higher stability of the LNPs at 4 °C after synthesis, without requiring freezing temperatures for their transport and storage, thus facilitating their distribution to clinical settings [47]. A study assessing the efficacy of formulated LNPs showed that LNPs remained stable for 15 days in storage at 4 °C after synthesis; although there was a reduction in activity within the first 24 h, the ability to transfect cells in vitro successfully was retained and remained unchanged for the remaining days [48]. This study also demonstrated rapid and highly efficient transfection of cells in vivo in rats and pigs, with peak expression within 20 h of delivery. The expression decreased to an almost negligible level by week 2 after administration, which renders this approach applicable for indications where transient expression is preferred or when repeated administration is feasible [48]. Non-viral vectors are less used for lung delivery. This is a path that remains to be further explored. Another advantage of using nanoparticles for the delivery of RNA-based gene therapy includes the enhancement of cellular uptake, endosomal escape, and protection from nuclease degradation; furthermore, targeted delivery can be achieved by enhancing nanoparticle compatibility through surface modifications. A study tested two polymers to deliver miRNA mimics into CF airway epithelial cells, including PEI and chitosan [49]. While PEI-based nanoparticles were more effective in facilitating miRNA uptake into the epithelial cells than chitosan, both nanoparticles appeared to be nontoxic [49]. Another study used an experimental PAH rat model to assess the therapeutic effects of the intravenous delivery of antisense oligonucleotide against miR-145 (anti-miRNA-145) with loaded LNPs injected three times over 5 weeks. The authors found that anti-miRNA-145 was distributed mainly in the lungs, liver, kidney, and spleen, and no major off-target effects were observed in rats [50]. A recent review summarized the delivery of miRNA by employing various types of nanoparticle-based therapeutics in respiratory diseases, including the common nanoparticles mentioned above and some novel nanoparticle delivery systems [51]. Apart from RNA-based therapies, decoy oligodeoxynucleotides (ODNs) were recently found to be another promising gene therapy agent, especially nuclear factor KB (NFKB) decoy ODNs, which have shown great potential in the treatment of respiratory diseases by reducing the NFkB-mediated inflammatory signaling pathway [52]. Various nanoparticles have been tested to deliver NFkB decoy ODNs both in vitro and in vivo with impressive outcomes. In an in vitro model of CF induced by interleukin-1β or *Pseudomonas* *aeruginosa* lipopolysaccharide-stimulated bronchial epithelial cell lines, NFkB decoy ODNs coated with polysialic acid-N-trimethyl chitosan or poly(D,L-lactide-co-glycolide), large porous particles efficiently reduced the secretion of multiple pro-inflammatory mediators of CF [53,54]. Moreover, in a PAH rat model, a single intratracheal instillation of polymeric nanoparticle (NP)-mediated NF-kB decoy resulted in the delivery of NPs into lungs and attenuated the setting of PAH by reducing vascular remodeling and inflammation [55]. More details about NFKB decoy ODN-based gene therapies in respiratory diseases were summarized in a recent review [52].

**Figure 2:**
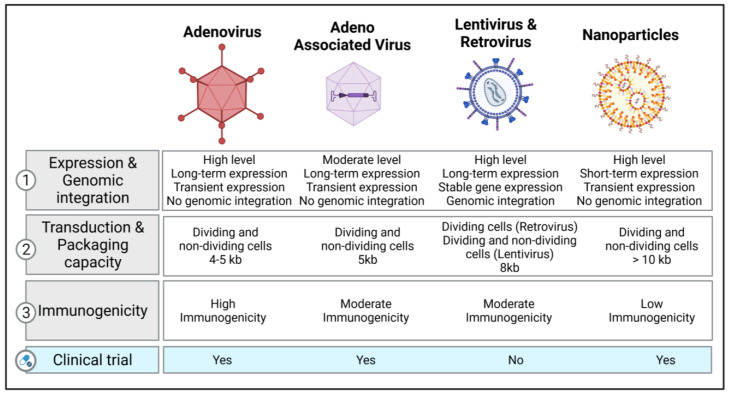
Vector platforms and their characteristics available for gene transfer. The table lists the different types of vectors (adenovirus, adeno-associated virus, lentivirus, retrovirus, and nanoparticles) used in clinical and preclinical studies for treating respiratory diseases and refers to specific information related to expression levels, genome integration, transduction, packaging capacity, immunogenicity, and their use in clinical trials. Created with BioRender.com (accessed on 3 March 2022).

Recent clinical studies have tested the options of non-viral therapy for respiratory diseases. On the basis of a preliminary clinical trial using the lipid GL67A as the vector, a phase 2B clinical trial was performed to evaluate the efficacy in CF patients, and it revealed a modest benefit on lung function after one year [56,57]. Another study tested DNA nanoparticles with PEG-substituted 30-mer lysine polymers in CF patients, reporting successful transduction efficacy without serious adverse effects [58]. Although the current studies show encouraging results, more studies are required. Figure 2 illustrates the different types of vectors that are currently used in clinical and preclinical studies with their respective characteristics.

## 3. Gene-Editing Strategies

Gene therapy approaches have focused on expressing exogenous DNA to correct a disease caused by a genetic mutation. Novel technologies are being developed to edit and repair mutated genes in vivo, insert new genes, or delete undesirable genes. Gene editing relies on homology-directed repair or non-homologous DNA ends joining, using cellular machinery of double-stranded breaks made in genomic DNA. Double-stranded breaks can be introduced in specific locations using nucleases such as transcription activator-like effector nucleases (TALENs) or CRISPR/Cas9 [25]. TALENs are proteins that combine a DNA-binding domain that recognizes specific DNA sequences with a Fokl endonuclease domain. The limitation of this system is the requirement that a new protein must be engineered for each DNA target site of interest.

### 3.1. CRISPR-Cas9 as a Genome-Editing Tool

CRISPR/Cas9 is a recently emerged gene-editing tools that involves using gene editing to guide RNAs that include complementary sequences to the target site. The formation of the heteroduplex complex between the guide RNA and DNA allows for cleavage by Cas9 nuclease, which makes it a simpler and more efficient tool for gene editing [59]. The main limitation of this system is the need for an adjacent protospacer motif and NGG sequence [60]. CRISPR/Cas9 can be used for the reversible epigenetic control of targeted genes. Enzymatically deactivated Cas9 can be fused to activator or repressor domains to activate or silence the gene expression of target genes, respectively [61,62]. Moreover, CRISPR/Cas9 is used for base editing. One nitrogenous base can be substituted without producing a double-stranded break by fusing a deactivated Cas9 with other deaminase enzymes [63].

Preclinical studies assessed the therapeutic efficacy of CRISPR/Cas in a number of diseases, including congenital genetic lung diseases (e.g., inherited surfactant protein syndromes and CF) [64,65], infectious diseases [66], lung cancer [67], and immunological diseases [68]. Moreover, recent studies tested CRISPR/Cas in clinical studies. The first clinical trial using CRISPR/Cas9 was carried out in the context of metastatic non-small-cell lung cancer to target the *PD-1* gene in T cells [69]. An analysis of 12 patients who received the treatment showed that the clinical use of CRISPR-Cas9 gene-edited T cells is safe [70]. Despite the potential benefits, more studies are urgently required to confirm the safety and therapeutic efficacy of the treatment. Currently, dozens of registered clinical trials with CRISPR-Cas are being carried out. Recently, CRISPR/Cas has been developed to detect SARS-CoV-2, which may provide more reliable test results than RT-qPCR [71,72].

### 3.2. Base- and Prime-Editing Technologies

Recent gene-editing strategies allow for the correction of genetic mutations with higher specificity, efficiency, accessibility to specific loci with lower off-target activity, and frequency of insertion/deletion at non-targeted sites [73]. Base editing is a CRISPR-Cas9-based genome-editing technology and represents a promising therapeutic approach for genetic disorders caused by point mutations [74]. It allows the precise and safe editing of DNA sequences at specific loci without inducing double-strand breaks. It was previously reported by Rees and Lui in 2018 that around 60% of the pathogenic point mutations could be potentially corrected by base editors (BEs) [75]. As a result, multiple BEs that allow base conversions have since been developed. For example, cytosine BEs convert a C:G to a T:A base pair and adenine BEs allow the conversion of an A:T into a G:C. BEs are guided by sgRNA to the locus of interest, and the d/nCas9 recognizes a PAM sequence. Prime editing is a novel method often described as a “search-and-replace” genome-editing technology [76]. It enables DNA substitutions, insertions, and deletions without requiring a PAM sequence adjacent to the targeted site. The prime-editing system relies on a prime-editing-extended guide RNA (pegRNA)-guided reverse transcriptase instead of a deaminase [74]. However, higher InDel frequency has been reported with prime editing than the CRISPR/Cas9 nuclease system in the base-editing method.

## 4. Applications of Gene Transfer for Respiratory Diseases

Respiratory diseases represent the most common medical conditions in the world [1]. Common factors underlying lung diseases are smoking, infections, allergens, and genetic disorders. The promise of gene therapy is well illustrated by the numerous preclinical studies and clinical trials for treating PAH, IPF, CF, asthma, COPD, AAT deficiency, NSCLC, and COVID-19.

### 4.1. Pulmonary Arterial Hypertension (PAH)

PAH is a multifactorial disease characterized, in part, by angioproliferative vasculopathy caused by the pulmonary vascular remodeling of the small pulmonary arteries. Sustained vascular remodeling is associated with arterial stiffening, thickening, and increased pulmonary vascular resistance (PVR) [77]. If left untreated, these vascular changes can lead to right ventricle (RV) failure and the death of the patient [78,79]. Most of the FDA-approved therapies currently available for PAH target the imbalance between vasoconstrictor and vasodilator agents and endothelial cell function. The conventional drugs include phosphodiesterase 5 inhibitors, endothelin receptor antagonists, cGMP activators as well as prostacyclin analogs, and receptor agonists [80]. Unfortunately, no curative treatment is available. This is partly due to the complex and multifactorial etiology of PAH. Indeed, several genes and signaling pathways are dysregulated in PAH [81,82,83,84,85,86,87]. As a result, gene therapy-mediated approaches have been extensively investigated.

#### 4.1.1. Bone Morphogenetic Protein Type 2 Receptor (BMPR2)

One of the most common pathomechanisms in PAH is an alteration of BMPR2 signaling. More than 70% of patients with hereditary PAH show heterozygous mutations in the *BMPR2* gene. Interestingly, idiopathic PAH patients also showed a strong reduction in *BMPR2* levels [4,88]. The loss of BMPR2 function or expression is associated with a severe hemodynamic profile and poor outcomes in PAH patients [89].

Previous studies demonstrated that the impairment of BMPR2 signaling contributes to a PAH-like phenotype [89]; thus, the modulation of BMPR2 signaling using gene transfers represents a promising treatment strategy. An intravenous injection of Ad vector containing *BMPR2* reversed PAH in mice carrying a *BMPR2* mutation, as shown by reduced RV systolic pressure (RVSP) and hypertrophy [90]. In the chronic hypoxia-induced PAH (CH-PAH) rat model, the administration of an Ad vector encoding *BMPR2* reduced the pulmonary artery and RV pressures, with vascular and ventricular remodeling [91]. Inconsistently, a different study found that the intratracheal delivery of an Ad vector containing the *BMPR2* gene did not improve the disease, despite a good distribution of the gene in the arteriolar network in the monocrotaline-induced PAH (MCT-PAH) model in rats [92]. Another study conducted in the same model showed that Ad-mediated *BMPR2* gene therapy on the endothelium using an intravenous injection significantly reduced RV hypertrophy and pulmonary arterial pressures, with a switch from TGF-β signaling to BMPR2 signaling, with the activation of the PI3K pathway, and decreased P38-MAPK signaling at the molecular level [93]. The different results from the two studies may suggest the importance of targeting the endothelium during *BMPR2* gene therapy. By similarly using the in vivo intratracheal delivery of an Ad vector containing the *BMPR2* gene, other studies successfully up-regulated the expression of *BMPR2* and restored SMAD-1/5/8 signaling in the pulmonary vasculature and reduced the proliferation of vascular cells in experimental PAH models [94,95].

#### 4.1.2. Sarco-Endoplasmic Reticulum Calcium-ATPase 2a (SERCA2a)

SERCA2a is a membrane transport protein ubiquitously expressed in the endoplasmic reticulum. SERCA2a regulates intracellular calcium dynamics and muscle contraction [96]. The dysregulation of *SERCA2a* expression and activity significantly impairs the intracellular levels of calcium and, subsequently, a broad panel of biological functions, including cell death, survival, and proliferation [97].

Previous studies showed that *SERCA2a* levels are decreased in rodent models of vascular injury and PAH [98]. The intratracheal delivery of lung-targeted *SERCA2a* gene transfer using an AAV1 attenuated MCT-induced PAH, as evidenced by reduced RVSP, pulmonary artery pressure (mPAP), and vascular remodeling. AAV1.*hSERCA2a* delivery also inhibited cardiac hypertrophy and fibrosis in MCT-induced PAH rats [99]. Additionally, the instillation of AAV1.*SERCA2a* improved myocardial electrophysiological remodeling and reduced susceptibility to ventricular tachyarrhythmia, demonstrating the therapeutic potential of SERCA2a gene delivery for arrhythmia suppression [100,101]. In addition, the nebulization of AAV1.*SERCA2a* attenuated structural changes and resistance in pulmonary arteries and pointed to better long-term survival in a pig model of chronic PAH [102]. In 2021, our group assessed the therapeutic efficacy of single and combination therapies using an AAV1 encoding human *SERCA2a* or *BMPR2* and a STAT3 inhibitor named HJC0152 in a rat model of severe PAH induced by unilateral left pneumonectomy combined with a single injection of monocrotaline (PNT/MCT) [95,103]. Our results demonstrated that the lung-targeted delivery of AAV1.*hSERCA2*, AAV1.*hBMPR2* alone, or HJC0152 significantly reduced mPAP and vascular remodeling, while improving RVSP and RV remodeling. Interestingly, a combination therapy using the STAT3 inhibitor improved the beneficial effects of SERCA2a and inhibited RV structural and functional changes. Mechanistically, here we found that the loss of *SERCA2a* repressed *BMPR2* expression via STAT3 overactivation [95].

#### 4.1.3. SIN3 Transcription Regulator Family Member A

The SIN3a complex is a molecular platform implicated in the regulation of a number of transcription factors and epigenetic modulators. The SIN3 complex consists of the SIN3a and SIN3b corepressors, histone deacetylase (HDACs), methyl-CpG-binding protein 2 (MeCP2), and other associated proteins that regulate the transcriptional machinery. SIN3a does not interact directly with DNA, but instead acts as a scaffold protein for several transcription factors. SIN3a promotes the recruitment of HDACs, which may suppress the expression of genes. Growing evidence suggests that SIN3a may also activate gene transcription and potentiate the expression of specific target genes, including *BMPR2* [104]. In 2021, our group demonstrated that *SIN3a* levels were significantly reduced in PAH patients as well as rodent models of PAH. The intratracheal delivery of AAV1.*hSIN3a* significantly lowered mPAP and RVSP. Our findings collectively demonstrated that restoring SIN3a levels in the pulmonary vasculature reversed PAH by limiting adverse hemodynamic profiles and RV remodeling, characterized by attenuated RVSP, PAP, and the Fulton index in the MCT-induced PAH and SuHx-induced PAH model [105]. The integration of an RNA-seq dataset revealed that SIN3a overexpression reduced the expression of DNA methyltransferase 1 (*DNMT1*) and enhancer of zeste homolog 2 (*EZH2*), while promoting the expression of Tet methylcytosine dioxygenase 1 (*TET1*), a key enzyme in DNA demethylation. *SIN3a* promoted *BMPR2* expression at the molecular level by decreasing H3K27me3 abundance and DNA methylation within the promoter region and reducing *CTCF* (CCCTC-binding factor) binding [105].

#### 4.1.4. Endothelial Nitric Oxide Synthase (ENOS)

Nitric oxide (NO) is an endogenous gas with potent cardiovascular protective effects synthesized by a group of enzymes known as nitric oxide synthases (NOS). Endothelial NOS (ENOS) is the main source of NO in the pulmonary circulation. NO is a strong pulmonary vasodilator that regulates pulmonary vascular tone, impedes platelet aggregation, and inhibits SMC migration and proliferation [106]. The impaired bioavailability of endothelium-derived NO and the decreased expression of ENOS contribute to the pathogenesis of PAH. A previous study from Janssens et al. found that aerosolized recombinant Ad containing constitutive *ENOS* reduced acute hypoxic pulmonary vasoconstriction in rat lungs [107]. Similarly, the aerosol delivery of inducible NOS promoted NO production in the lungs and significantly reduced hypoxia-induced PAH in rats [108]. Additionally, the intravenous administration of endothelial progenitor cells (EPC) overexpressing *ENOS* attenuated MCT-induced PAH. The Pulmonary Hypertension and Angiogenic Cell Therapy trial (a first-in-human trial) conducted using 7–50 million *ENOS*-transfected EPCs, which were delivered into the right atrium via a multiport pulmonary artery catheter, showed that the infusion was well tolerated. The authors found that the administration of *ENOS*-EPC seems to lower pulmonary vascular resistance during the 3-day delivery period, while no sustained hemodynamic improvements were reported at 3 months [109].

#### 4.1.5. Voltage-Gated Potassium Channel Kv1.5

Potassium voltage-gated channel, shaker-related subfamily, member 5, also known as KCNA5 or *KV1.5*, plays a critical role in the regulation of the membrane potential of pulmonary vascular cells and vascular tone [110]. Previous studies showed that hypoxia inhibits the activity of KV1.5 and increases cytosolic Ca^2+^ concentration in PASMCs. Kv1.5 participates in the pathogenesis of PAH by inducing a sustained depolarization and increasing the intracellular calcium and potassium concentrations, thus modulating cell proliferation and apoptosis [111]. Similarly, acute hypoxia in animal models of PAH also inhibited Kv1.5 channel activity [112]. *KV1.5* overexpression in rat PASMCs decreased apoptosis resistance by increasing caspase-3 activity [113]. In vivo, the nebulization of Ad5 encoding human *KV1.5* (Ad5-GFP-*KV1.5*) restored O2-sensitive whole-cell K+ current and increased PASMC current density in PASMCs, normalized hypoxic pulmonary vasoconstriction, reduced PVR, and significantly improved cardiac output. As a result, the in vivo gene delivery of Ad5-GFP-*KV1.5* also attenuated RV hypertrophy and PA medial hypertrophy in a chronic hypoxic PAH rodent model [114].

#### 4.1.6. Survivin

Survivin is described as an “inhibitor of apoptosis” and is downregulated in the pulmonary vasculature in PAH [115]. Therapeutic strategies include gene transfer to restore survivin expression and re-sensitize cells to apoptosis. Previous studies showed that an inhaled Ad vector encoding a dominant-negative survivin mutant reversed MCT-induced PAH and increased survival by 25% [115]. Dominant-negative *SURVIVIN* gene transfer also decreased PASMC proliferation and apoptosis resistance in pulmonary arteries, decreasing medial muscularization, PVR, and RV hypertrophy. The authors further demonstrated that the inhibition of survivin induces the depolarization of the mitochondrial membrane and cytochrome c efflux in the cytoplasm, potentiates the nuclear translocation of apoptosis-inducing factors, and increases Kv channel current [115]. Evidence suggests that Kv channel activation can induce apoptosis and decrease proliferation in various tumor cells [116].

#### 4.1.7. Vasoactive Intestinal Peptide (VIP)

VIP is a small peptide that belongs to the glucagon growth hormone-releasing superfamily. VIP reduces the proliferation of PASMCs isolated from PAH patients [117]. Treatment with aerosolized *VIP* was shown to improve hemodynamics and prognostic parameters in a prospective, controlled intraindividual clinical study with eight PAH patients [117]. Alterations in the *VIP* gene have been identified in idiopathic PAH patients, and the loss of *VIP* can induce moderate PAH in mice [118,119]. *VIP* gene transfer may represent a promising alternative for treating PAH patients carrying a mutation in the VIP gene. In vitro, *VIP* overexpression using an Ad reduced proliferation in rat PASMCs [120].

#### 4.1.8. Calcitonin Gene-Related Peptide (CGRP)

Calcitonin gene-related peptide (CGRP) is expressed in the lung’s nerves and endocrine cells, while its receptor is highly expressed in PASMCs [121]. Given its antiproliferative properties, several studies have evaluated the potential of *CGRP* gene transfer for treating PAH. Gene delivery of *CGRP* reduces cell proliferation in vitro [122]. The intratracheal administration of an Ad vector containing the pre-pro *CGRP* gene can attenuate PAH in the CH-PAH mouse model, with reduced PVR, RV hypertrophy, and pulmonary vascular remodeling [123,124]. Moreover, the transplantation of EPCs overexpressing *CGRP* can attenuate established PAH in a rat model induced by abdominal aorta to inferior vena cava shunt in immunodeficient rats [125].

The following Table 1 recapitulates the main therapeutic genes used for treating pulmonary hypertension (PH) in preclinical studies.

### 4.2. Idiopathic Pulmonary Fibrosis (IPF)

IPF is a serious interstitial lung disease associated with the aberrant accumulation of fibrotic tissue in the lung parenchyma. Previous studies showed that risk factors, such as genetic mutations, tobacco smoke, and viral infections, increase susceptibility to IPF. Altered folding and the processing of surfactant proteins may induce a pathological endoplasmic reticulum stress response, increase oxidative stress, and induce DNA damage. Although the pathophysiology of IPF is not completely understood, chronic injury of alveolar epithelial type II cells (AECII) is described as an essential hallmark of the disease. The excessive apoptosis of AECII disrupts epithelial homeostasis and dysregulates the regenerative capacity and epithelial–mesenchymal interactions [126]. Few FDA-approved drugs are available for treating IPF. Pirfenidone is a pleiotropic molecule that inhibits collagen synthesis and fibroblast proliferation, which is now available in more than 60 countries, including 27 EU countries and the United States. Meanwhile, combination therapy using prednisone with immunomodulatory agents such as azathioprine was shown to be ineffective. Recent data from a randomized controlled trial suggested that the combination therapy with prednisone, azathioprine, and the antioxidant acetylcysteine increased the risks of death and hospitalization in IPF patients. Consequently, the study was prematurely interrupted owing to safety concerns associated with the three-drug regimen [127].

#### 4.2.1. SERCA2a

Calcium homeostasis is essential in the regulation of gene expression in human pulmonary fibroblasts. The impairment of calcium signaling dysregulates myofibroblast differentiation as well as their synthesis and secretion function. In 2015, Mukherjee et al. showed that nifedipine, a calcium channel blocker, inhibited BLM-induced IPF in mice by improving lung function and lung gas exchange. Nifedipine treatment prevented extracellular matrix deposition and decreased soluble collagen and hydroxyproline content. More recently, our group identified a drastic decrease in *SERCA2a* levels in IPF patients and bleomycin (BLM)-challenged mice [128]. The intratracheal nebulization of AAV1.*SERCA2a* effectively reduced lung fibrosis and vascular remodeling and improved gas exchange [128,129]. Interestingly, the local delivery of AAV1.*hSERCA2a* also attenuated RV pressures and hypertrophy in bleomycin-induced PF [128]. Gene therapy with AAV1.*SERCA2a* also increased the lifespan by 45% compared to BLM mice treated with a control AAV encoding luciferase [128]. In vitro, *SERCA2a* overexpression inhibited fibroblast proliferation, the expression of fibrosis markers, and the differentiation to myofibroblasts by blocking the OTUB1/forkhead box M1 (FOXM1) and promoting the SnoN/Ski axis [128].

#### 4.2.2. Caveolin-1

Caveolin-1 is a major component of caveolae that is highly expressed in the lungs, alveolar epithelial type 1 cells, endothelial cells, fibroblasts, and leukocytes [130]. CAV-1 regulates many signaling pathways, including MAPK and P13K, resulting in cell growth suppression and the induction of apoptosis. Previous studies suggest that CAV-1 may regulate fibrosis by regulating TGF-beta signaling, ECM production, and inflammation [131]. For example, Wang et al. identified, in 2016, a marked reduction of Cav1 expression in IPF patients and BLM mice. In vivo, the authors demonstrated that the intratracheal administration of Ad.*CAV1* attenuated BLM-induced pulmonary fibrosis by suppressing TGF-β1-induced ECM production in pulmonary fibroblasts and modulating TGF-β1-induced SMAD signaling and COL1A1 production via the ERK1 pathway. Concomitantly, this study also showed that Ad.*CAV1* blocks TGF-β1-induced fibronectin production via the JNK1 pathway [132].

#### 4.2.3. SMAD Family Member 7 (SMAD7)

TGF-β signaling plays a key role in the pathogenesis of tissue fibrosis. The activation of TGF-β receptors potentiates the phosphorylation of Smad2 and Smad3, which leads to the formation of hetero-oligomeric complexes with Smad4. In response to TGF-β stimulation, the nuclear translocation of the Smad4 complex participates in gene expression regulation. Smad7 is well described as an antagonist of TGF-β signaling by interfering with the activation of Smad2 and Smad3. The intratracheal administration of a recombinant Ad vector carrying *SMAD7* cDNA reduced type 1 pre-collagen mRNA levels, attenuated hydroxyproline content, and inhibited bleomycin-induced Smad2 phosphorylation in the mouse lungs. Furthermore, Ad.CMV-*SMAD7* prevented bleomycin-induced lung fibrosis. However, *SMAD7* overexpression with Ad did not inhibit BLM-induced pulmonary inflammation and TGF-β production in mouse lungs [133].

#### 4.2.4. Telomerase Reverse Transcriptase (TERT)

Telomerase is a ribonucleoprotein polymerase that regulates the telomere length by adding the repeat sequence TTAGGG at the 3’ end. The ribonucleoprotein telomerase has a core component composed of a reverse transcriptase (TERT) subunit and a separately coded RNA template (TERC or TR), which, along with a series of associate proteins, leads to the extension and replenishment of telomeres [134]. This is essential for chromosome end protection and genome stability [135]. The dysregulation of the telomerase expression through *TERT* promoter mutations is associated with increased cell proliferation, apoptosis resistance, impaired differentiation, and senescence [136]. In 2018, Povedano et al. used AAV9 vectors to assess the efficacy of *TERT* gene transfer in the low-dose BLM-induced IPF model. First, the authors found that intravenous delivery of AAV9.*TERT* predominantly transduced AECII cells [137]. Importantly, *TERT* gene transfer decreased inflammation and inhibited fibrosis 1–3 weeks after delivery. *TERT* gene therapy is associated with longer telomeres, increased cell proliferation, lower DNA damage, apoptosis, and senescence [137]. A transcriptome analysis of AECII cells transduced with TERT further confirmed the inhibition of fibrosis and inflammation pathways [137].

### 4.3. Cystic Fibrosis

Cystic fibrosis (CF) is an inherited disorder characterized by severe damage to the lungs, digestive system, and other organs. Cells located in the passageways of the lungs, pancreas, and other organs produce thick mucus with concentrated mucins, causing respiratory failure along with systemic obstructions and abnormalities [138]. Therapeutic approaches that include mucolytic agents, physiotherapy, exercise, mannitol, or hypertonic saline aim to manage symptoms by breaking down the mucus buildup, improving airway clearance, or rehydrating the airway surface. Unfortunately, there is currently no cure for cystic fibrosis. Ultimately, a lung transplant may be the only therapeutic alternative as the disease progresses [139].

#### Cystic Fibrosis Transmembrane Conductance Regulator (CFTR)

The *CFTR* gene encodes for an ABC transporter class ion channel protein that conducts chloride ions across epithelial cell membranes. CFTR regulates the flow of salt and fluids in and out of the cells across the body, especially through the epithelial cell membranes. Mutations in the *CFTR* gene play a central role in the pathogenesis of CF. Therefore, mutations within the *CFTR* gene impair the function of chloride channels and ultimately dysregulate the flow of chloride ions and water across cell membranes. Major pulmonary abnormalities have been identified in CF patients with *CFTR* loss-of-function mutations [140]. More than 2000 variants have been reported in the *CFTR* gene, and more than 400 are known to cause disease [141]. The second most prevalent nonsense variant in the *CFTR* gene is W1282X (c.3846G > A), which contains premature termination codons, which may be degraded via nonsense-mediated mRNA decay [141]. Gene replacement therapies that deliver correct copies of a functional gene to restore CFTR activity into affected CF airway cells represent a promising therapeutic strategy for preventing and treating CF lung disease [142].

Conducted in 1993, the first clinical trial using Ad-mediated gene therapy for treating CF failed to restore *CFTR* expression in nasal epithelia of CF patients [143,144,145,146]. New Ad- and AAV-based vectors have since been developed and have shown encouraging results to restore *CFTR* expression and activity into airway epithelial cells. For example, Cooney et al. used a piggyback/Ad.*CFTR* vector to phenotypically correct CF airway disease in pigs. This method combines the delivery efficiency of an Ad-based vector with the persistent expression of a DNA transposon-based vector. Aerosolized piggyBac/Ad showed a widespread pulmonary distribution of the vector and efficiently restored functional CFTR with a single vector administration [147]. One of the main limitations of AAV is the capacity of DNA packaging, which requires the truncation of the *CFTR* gene. Additionally, because AAV vector genomes are not integrated into the host genome, AAVs produce only transient effects that are rapidly lost with airway cell turnover [148].

Because lentiviruses can transduce dividing and non-dividing cells, their use opens new avenues for gene addition strategies for treating CF. [142]. Importantly, they can also be manipulated and optimized to define the tissue and cell tropism by altering surface receptor recognition elements. Furthermore, their integration into the host genome provides long-term benefits. In 2017, Alton et al. developed a more efficient lentiviral gene vector and showed that rSIV.F/HN-pseudotyped lentiviral vector administration to murine lungs significantly restored *CFTR* expression and function with a 90–100% transduction efficiency by employing clinically relevant delivery devices. In recent years, nanoparticles have been further investigated for *CFTR* gene therapy to deliver *CFTR* mRNA directly and improve chloride channel function in CF mice, with a better response compared to liposomal delivery. However, due to the acute action and nanoparticle stability, further optimization is required and should be considered in future studies [149]. In 2015, a monthly repeat-dose phase II *CFTR* non-viral (liposome) gene transfer clinical trial showed modest and transient lung function benefits in CF patients [57]. It successfully demonstrated that the nebulization of non-viral *CFTR* gene therapy is a safe and efficient strategy for treating CF with low immunogenicity.

In addition to gene replacement therapies, several recent studies have employed CRISPR gene-editing tools to permanently correct the genetic defects caused by the W1282X-CFTR mutation. In 2020, Vaidyanathan et al. used CRISPR/cas9 in vivo to successfully correct the F508 mutation in the *CFTR* gene in primary airway stem cells obtained from CF patients [150]. This selection-free strategy significantly restores the CFTR function in differentiated epithelial sheets. This study offers new opportunities for correcting genetic disorders in CF and provides a promising means with which to optimize the transplantation of corrected basal stem cells into the upper airway to treat CF disease in future studies. The successful optimization of stem cell transplantation into the sinuses may trigger further investigations for treating CF lung disease [150]. More recently, Santos et al. compared the capacity of HDR mediated by Cas9 and Cas12a to correct the W1282X-*CFTR* mutation and found that Cas9 had a ~2.3-fold higher percentage of precise editing than Cas12a and a lower indels frequency [151]. Importantly, increased *CFTR* mRNA and CFTR protein expression were associated with the restoration of CFTR function in Cas9-edited cells [151]. Collectively, this study successfully demonstrated the therapeutic potential of gene editing for patients carrying the W1282X-*CFTR* variant. Another study used the antisense oligonucleotide for splicing modulation to enhance the specific retention or skipping of exon 23 of the *CFTR* transcript to eliminate the W1282X mutation [152]. Ultimately, specific skipping over exon 23 helped to overcome RNA degradation induced by the nonsense-mediated mRNA decay mechanism and allowed the production of partially active CFTR proteins [152]. In this study, the authors demonstrated that the antisense oligonucleotide (SPL23–2 and SPL23–3) restores the CFTR function in primary human nasal epithelial cells from a patient homozygous for W1282X, also known as 16HBEge cells [152]. Similarly, Kim et al. developed a cocktail of two antisense oligonucleotides (i22-3ss and i23-5ss) for exon skipping that allow for gene-specific nonsense-mediated messenger RNA (mRNA) decay evasion [153]. The specific deletion of exon 23 preserves the reading frame and produces a functional truncated CFTR protein, which was sufficient to restore CFTR-mediated chloride current in human bronchial epithelial cells [153].

### 4.4. Asthma

Asthma is a chronic lung disease characterized by inflamed and narrowed airways, which ultimately alter pulmonary compliance and increase respiratory effort. Importantly, it is now well established that type 2 immune mechanisms drive the inflammation in about 50% of asthmatic patients. In asthmatic patients, immune hypersensitivity involves IgE antibodies, which bind mast cells with high affinity when a pollutant or allergen is inhaled. Airway hyperresponsiveness may be due to histamine release from mast cells or increased airway smooth muscle mass. The number of myofibroblasts causes an increase in epithelium, which narrows the smooth muscle cell layer. As a result of the increased thickening of the basement membrane, a person can have irreversible obstruction to airflow, which is believed to be caused by airway remodeling [154]. Current treatments for asthma rely mainly on three different pharmaceutical categories: “controller” to reduce inflammation, “reliever” to prevent exercise-induced bronchoconstriction, and an “add-on” used in patients with severe and persistent symptoms. Asthma therapy includes corticosteroids and a short-acting beta-2-agonist (SABA). Although traditional therapies attempt to manage the symptoms, the underlying pathophysiology and pathobiology of asthma are not targeted [155].

#### 4.4.1. GATA-Binding Protein 3 (GATA3)

GATA3 is a zinc finger transcription factor that belongs to the GATA family and recognizes the consensus recognition motif WGATAR (W = A or T and R = A or G). GATA3 has been identified as a master regulator for cell differentiation of the T helper type 2 (Th2) cytokines. *GATA3* expression is significantly up-regulated in asthmatic airways and strongly correlates with IL-5 levels and airway hyperresponsiveness (AHR) [156]. Emerging strategies aim to impair *GATA3* expression using different strategies. DNAzyme is a new class of antisense molecules with inherent enzymatic activity that binds to and cleaves target RNA.

The intranasal application of a *GATA3*-specific DNAzyme, gd21, on 4 subsequent days decreased the number of eosinophils and mucus-producing goblet cells in bronchoalveolar lavage in an acute model of experimental allergic asthma [157]. Antisense GATA-3 inhibits the cellular inflammatory response and improves airway hyperresponsiveness to methacholine in models of acute allergic airway inflammation. Histological analysis showed the reduced infiltration of inflammatory cells. The safety and tolerability of a new inhaled *GATA3*-specific DNAzyme, SB010, has been investigated in patients with TH2-driven asthma. The phase I trial for SB010 was conducted on 108 subjects and revealed that SB010 is safe and well tolerated in asthmatic patients. Given the good safety profile of this agent, a phase IIa proof-of-concept study is currently being pursued in asthmatic patients [158]. Preclinical and clinical studies that evaluated a new human *GATA3* DNAzyme candidate, called hgd40, have also shown promising results [159]. Hgd40 inhalation targeted most cell types in the lung and airway and significantly silenced *GATA3* expression [159]. Thus, levels of type 2 cytokines were drastically decreased while the Th1-derived cytokine IFN-gamma was potentiated. Overall, hgd40 ameliorated eosinophilia, mucus production, and tissue remodeling [159].

#### 4.4.2. Thymulin (Formerly Called “Serum Thymus Factor” or FTS)

Thymulin FTS (serum thymus factor) is a biologically inactive nonapeptide that becomes active when coupled with zinc ions in vivo [160]. FTS is associated with anti-inflammatory and anti-fibrotic properties in various disease models [161]. In 2014, da Silva and collaborators demonstrated that DNA nanoparticle-mediated *Thymulin* gene therapy prevented airway remodeling in an experimental model of allergic asthma [162]. Nanoparticle-based technologies relying on highly compacted DNA nanoparticles with CK_30_PEG have shown impressive results. These DNA nanoparticles were stable for at least 2 years at 4 °C and were non-toxic, non-inflammatory, and non-immunogenic. Previous studies demonstrated that nanoparticles carrying thymulin plasmid provided prolonged retention of upwards of 27 days post-administration in mice lungs [162]. A single dose of DNA nanoparticles effectively prevented lung remodeling processes in a rodent model of chronic allergic asthma, as demonstrated by decreased pulmonary inflammation, collagen deposition, and smooth muscle hypertrophy, and improved lung mechanics such as lung static elastance and airway hyperresponsiveness [162]. This study also provides a new platform on which to further develop nanoparticle-based gene therapies for asthma [162].

### 4.5. Chronic Obstructive Pulmonary Disease (COPD)

Chronic obstructive pulmonary disease (COPD) refers to a group of lung diseases associated with sustained airflow obstruction, leading to pulmonary heart disease and respiratory failure [163]. This chronic inflammatory lung disease is a leading cause of morbidity and mortality globally. The two main most common lung conditions for COPD are chronic obstructive bronchitis and emphysema. Besides genetics, risk factors include several environmental factors, such as cigarette smoking, air pollution, and chronic exposure to lung-irritating chemicals [164]. Other factors, such as age, and pre-existing conditions, such as asthma, airway hyper-responsiveness, chronic bronchitis, and infections, contribute to the development of COPD [163]. Current COPD treatments primarily target symptoms associated with airway obstruction and aim to improve breathing and the overall quality of life of patients with COPD [165]. They include anticholinergics, dual β2-dopamine 2 receptor antagonists, corticosteroids for their bronchodilator, and anti-inflammatory properties. In addition, non-pharmacological treatments, such as pulmonary rehabilitation therapy, oxygen therapy, ventilatory support, and surgical intervention (e.g., bullectomy, lung volume reduction surgery, and transplantation), are available and may help improve arterial oxygen tension and arterial oxygen saturation in patients [165]. However, there is no cure for treating COPD. COPD is a complex disease that results from multiple complex interactions between environmental factors and genetic disorders. Recent genetic evidence reported several polymorphisms in protease (e.g., MMP1 and MMP12), anti-protease (e.g., α1-antitrypsin, α1-antichymotrypsin, and α1-macroglobulin), antioxidant (e.g., heme oxygenase-1), cytokines (e.g., TGFβ1, TNF-α, IL-1 complex, IL-8, and IL-13), and other various genes (e.g., CFTR, human leukocyte antigen, vitamin D-binding protein, and β2 adrenergic receptor) that may be associated with COPD [166]. However, their implication and role remain to be further explored to evaluate the potential of genetic intervention for treating COPD. For example, previous studies reported that cigarette smoking is associated with decreased CFTR levels and mucus clearance defects in patients with COPD, suggesting that CFTR gene transfer may be a new therapeutic strategy [167]. The complex etiology of COPD and the lack of suitable animal models have significantly impacted the preclinical evaluation of gene transfer for treating COPD.

### 4.6. Alpha-1 Antitrypsin Deficiency (AATD)

AATD is a common inherited disorder associated with mutations in the *SERPINA1* gene. Loss of *SERPINA1* function is associated with an increased risk of lung diseases (emphysema, asthma, and chronic bronchitis) and/or liver diseases (hepatic failure, hepatitis, hepatomegaly, and cirrhosis) [168]. Alpha-1 antitrypsin (AAT) is a serum serine protease inhibitor whose function is to protect the lung from the activity of proteolytic enzymes, such as neutrophil elastase, trypsin, thrombin, and bacterial proteases. Low serum concentrations of AAT are characterized by an imbalance between proteases and AAT in the lung, contributing to lung parenchyma injury [169]. Environmental factors, such as cigarette smoking and long-term exposure to dust and chemicals, are known to worsen the severity of AATD. Over 100 variants of the *SERPINA1* gene have been reported and are known to be associated with AATD. The most common missense single-nucleotide polymorphisms associated with AATD include the dysfunctional S variant (Glu264Val) and Z variant (Glu342Lys) [170]. The first study to use the adenovirus-mediated transfer of a recombinant SERPINA1 gene to the lung epithelium in vivo to restore serum concentration in AAT in rats was conducted by Rosenfeld et al. in 1991 [171]. The authors demonstrated that the intratracheal delivery of a recombinant gene increased AAT synthesis and secretion by lung tissue. Recently, researchers have used AAV-based gene therapy to restore *AAT* expression at therapeutic levels. Different serotypes (AAV1, AAV2, and AAVrh.10) and routes of delivery (e.g., intravenous/intraportal vein, intramuscular, intrabronchial, and intrapleural) have been evaluated [172,173,174,175,176]. Although three AAV vectors have moved to clinical studies, further improvements in the vector design and delivery methods are critical to restore therapeutic AAT concentrations.

### 4.7. Non-Small-Cell Lung Cancer (NSCLC)

About 85% of lung cancers are non-small-cell lung cancer (NSCLC) and include different subtypes, such as adenocarcinoma and large-cell and squamous cell carcinoma. NSCLC remains the leading cause of cancer deaths in the world [177]. Genetic alterations affect both oncogenes and tumor suppressor genes. They regulate critical biological processes associated with tumorigenesis, such as self-sufficiency in growth signals, cell death, proliferation, angiogenesis, invasion, cell metabolism, and inflammation [177]. Analysis of whole-genome sequencing datasets revealed that the most common genetic alterations are located in tumor suppressor genes, such as the tumor suppressor p53 and the tumor suppressor candidate 2 (TUSC2) [178]. Thus, a number of gene therapy strategies for gene replacement have rapidly been developed to restore their expression and increase the sensitivity to conventional treatments, such as chemotherapeutic agents or radiotherapy [178].

#### 4.7.1. Tumor Suppressor p53

The first study demonstrating that the restoration of p53 expression could suppress tumor growth was conducted in 1994 by Fujiwara et al. using a retroviral vector in an orthotropic human NSCLC model expressing a mutant *P53* [179]. Several other preclinical studies have used an adenoviral vector (Ad-*P53*) and found that *P53* gene transfer promotes apoptosis and inhibits proliferation in NSCLC cell lines [180,181,182]. Using NSCLC orthotropic mouse models, p53 gene therapy enhances chemotherapy effectiveness and inhibits tumor growth by regulating pro-apoptotic signals, angiogenesis, and immune response.

The first clinical trial implementing *P53* gene transfer used a retroviral vector encoding the wild-type *P53* gene [183]. Intratumoral injections were performed in nine unresectable NSCLC patients that were unresponsive to conventional therapy. Three of the nine patients showed evidence of tumor regression, demonstrating the therapeutic potential and safety of *P53* gene replacement therapy, as no vector-related toxic effects were reported [183]. Given the potential for clinical application, a dose escalation trial was conducted in 28 NSCLC patients whose conventional therapy failed [184]. Successful gene transfer via intratumoral injection was confirmed by PCR analysis in 18 patients with evaluable post-treatment biopsy specimens [184]. However, vector-specific *P53* transcript levels were detected only in 12 patients using reverse transcription PCR analysis. Apoptosis was observed in most patients expressing vector-induced *P53*. A more than 50% tumor reduction was observed in 2 patients, disease stabilization in 16 patients, and disease progression in 7 patients [184]. Another study conducted at MD Anderson Cancer Center evaluated the degree of toxicity and antitumor activity in the delivery of Ad-*P53* gene therapy via bronchoscopic intratumoral injection in 12 patients with endobronchial NSCLC and expressing a *P53* gene mutation [185]. Their results showed that 6 of the 12 patients had significant improvement in airway obstruction, with 3 patients meeting the criteria for partial response. In 23 patients with bronchioloalveolar carcinoma, 16 patients showed evidence of disease stabilization, despite limited vector distribution [185].

#### 4.7.2. Tumor Suppressor Candidate 2 Gene (TUSC2)

Loss of tumor suppressor candidate 2 (TUSC2) expression or function has been observed in 80% of lung tumors [186] and is associated with significantly worse overall survival. The intratumoral administration of liposomal nanovesicles containing a *TUSC2*-expressing plasmid vector demonstrated potent antitumor activity by significantly reducing tumor growth in a subcutaneous H1299 and A549 lung tumor xenograft model in mice. Furthermore, intravenous injections of nanovesicles encapsulating the *TUSC2* gene significantly decreased the number of metastatic tumor nodules and improved survival in mice bearing experimental A549 lung metastasis [187]. An analysis of *TUSC2* expression showed the efficient distribution of *TUSC2* in the tumor, demonstrating the potent tumor-suppressive activity of TUSC2 in primary and disseminated human lung cancer. A dose escalation clinical trial (NCT00059605) was conducted 10 years ago to assess the toxicity of *TUSC2* nanovesicles after intravenous delivery and to determine the highest tolerated dose in 31 stage IV NSCLC patients with recurrent or metastatic NSCLC who were previously treated with platinum-based chemotherapy [188]. The authors found that *TUSC2* nanovesicles can be safely administered intravenously in lung cancer patients to restore the *TUSC2* levels in human primary and metastatic tumors and potentiate anti-tumor effects. Several phase II clinical trials (NCT01455389 and NCT05062980) are now being conducted to further evaluate the therapeutic potential of combination therapies using liposomal nanoparticles encapsulating a *TUSC2* (GXP-001) with pembrolizumab or erlotinib in patients with NSCLC.

### 4.8. Coronavirus Disease (COVID-19)

COVID-19 is an infectious disease caused by the novel severe acute respiratory syndrome coronavirus 2 (SARS-CoV-2) [189]. In just 2 years, the World Health Organization has already reported more than 428 million confirmed cases of COVID-19 worldwide, and the death toll had risen to more than 5,928,743. Clinical manifestations of the disease range from mild to critical and are associated with respiratory tract infections causing flu-like symptoms, leading to acute respiratory distress syndrome and respiratory failure [189]. While patients usually only show mild symptoms, some may develop severe cardiovascular complications, including coagulopathy, heart failure, acute coronary syndrome, and coronary artery aneurysm [190,191]. In response to the pandemic, modified RNA-based vaccines encoding an optimized SARS-CoV-2 full-length spike protein antigen have been rapidly developed to improve the immune response. These new vaccines are around 95% effective at preventing severe illness and COVID-19-associated hospitalization and death [189]. Ongoing epidemiological and pharmacovigilance studies are currently being conducted to evaluate vaccine safety and investigate clinical significance comprehensively.

**Figure 3:**
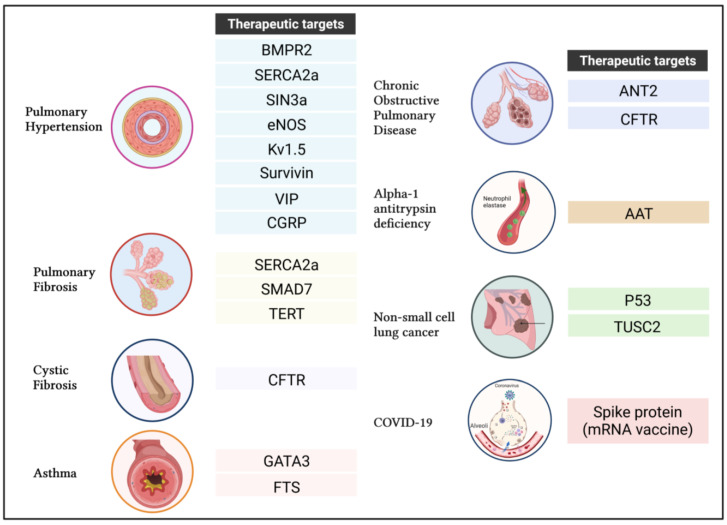
Main therapeutic candidates currently under investigation as lung-targeted gene therapies. Here, we provide examples of therapeutic candidates for gene therapy with substantial advances for the treatment of respiratory diseases, such as pulmonary hypertension, idiopathic pulmonary fibrosis, cystic fibrosis, asthma, chronic obstructive pulmonary disease, alpha-1 antitrypsin deficiency, non-small-cell lung cancer, and COVID-19. Created with BioRender.com (accessed on 3 March 2022).

Figure 3 recapitulates the main therapeutic candidates that are being investigated for treating the previously described respiratory diseases.

## 5. Limitations of Gene Therapy

Previous studies showed promising results in animal models using lung-targeted gene therapy, while no significant benefit was observed in clinical trials. The failure to translate preclinical findings to human studies can be attributed, at least in part, to the animal modeling itself, which fails to accurately replicate the exact features of human diseases. Importantly, increasing evidence shows that species-specific differences, including physiological barriers and immune responses, may severely impair the effectiveness of gene delivery in humans. Significant differences in lung cell biology across various species may explain, at least in part, why gene therapy has shown controversial results in experimental models of respiratory diseases in rodents when compared to human studies. Other factors related to patient selection (age and sex) and degree of disease severity at the time of treatment also influence treatment outcomes.

Because the lung epithelium is located at the interface of the human body with the inhaled environment, many defensive mechanisms attempt to maintain the integrity of the epithelial border. This defense response may target inhaled foreign particles and, thus, limit gene vectors’ ability to reach the epithelium in sufficient concentrations. Robust innate and acquired immune systems may prevent successful gene transfer. For example, alveolar macrophages phagocytose foreign particles and function as antigen-presenting cells to stimulate the host immune system, which leads to inflammation. In addition, the adaptive immune system produces neutralizing antibodies and cytotoxic T cells, which are activated in response to viral vectors after repeated administration. Typically, humans produce antibodies against Ad2, Ad5, AAV2, and AAV5, limiting their use in clinical studies; however, developing non-primate serotypes may partially circumvent this issue. Finally, it is worth mentioning that most lung diseases are associated with abnormal mucus production and inflammation, which may further reduce gene transfer efficacy. Indeed, the mucous layer that coats the epithelium traps and clears material through the mucociliary clearance system, which may also attenuate the effectiveness of inhaled gene therapy. In addition, the apical side of epithelial cells contains abundant glycoproteins and carbohydrates that bind viral particles and may impair their ability to reach the epithelium. This is particularly challenging since the receptors for adenoviral vectors are located on the basolateral side of epithelial cells, thus limiting their effectiveness. The transduction efficacy of AAV, such as AAV2, may also be impaired due to their structure.

Repetitive administration may be needed to achieve sustainable results, which has the added challenge of anti-vector immunity. To avoid repetitive administration, further investigation in stem cells or progenitor cells may provide promising options for future gene therapy studies along with new recombinant vectors.

## 6. Future Prospects

There is a growing body of evidence showing the benefits of gene therapy for lung diseases, and this motivates further study of these approaches. Understanding the limitations, along with the development of tools and approaches to overcome the barriers encountered, is critical to achieve improvement in the success rate. Novel and improved vectors that can avoid degradation and bypass the immune response after delivery [192,193] and gene-editing techniques of greater efficiency and precision [194], along with the development of disease models that are of high fidelity and which are species-specific [195,196], will contribute to advances in the field of gene therapy for respiratory diseases. Furthermore, the easy access to the airways and respiratory epithelial surface, through procedures that are either non-invasive (intranasal aerosolization) or minimally invasive (intratracheal aerosolization or bronchoscopy), is an advantage that can be exploited in the design of gene transfer approaches to the lung. It is expected that all these factors will contribute to higher transduction and transfection rates, efficient expression with limited off-target effects, and a long-term safety profile.

## 7. Conclusions

Gene therapy for respiratory diseases is a growing field with vast potential. The advances in genetic testing have unveiled the presence of altered gene expression profiles in several respiratory diseases. With a better understanding of the mechanisms underlying respiratory diseases, multiple potential therapeutic targets have been identified. Targeting different genes and employing diverse techniques, the data indicate promising results for treating PAH, IPF, CF, asthma, COPD, AAT deficiency, and NSCLC. Further advances in the field may bring a cure to diseases for which only palliative or suboptimal therapies are available.

## Figures and Tables

**Table 1:** Gene therapy in pulmonary hypertension. Table 1 displays the main therapeutic genes used for treating pulmonary hypertension (PH) in preclinical studies. Here, we show the PH models, type of vector, delivery methods, and reported results.

Gene	PH Models	Vector	Delivery Methods	Results	References
*BMPR2*	Chronic hypoxia Monocrotaline Sugen/hypoxia Pneumonectomy/monocrotaline	Adenovirus AAV1	Intravenous Intratracheal	↓ RVSP ↓ mPAP ↓ RV hypertrophy ↓ Vascular remodeling	[90,91,92,93,94,95]
*SERCA2A*	Sugen/hypoxia Monocrotaline Pneumonectomy/monocrotaline Pulmonary vein banding	AAV1	Intratracheal	↓ RVSP ↓ mPAP ↓ RV hypertrophy ↓ Vascular remodeling ↑ Cardiac function ↓ PVR	[95,99,100,101,102,103]
*SIN3A*	Sugen/hypoxia Monocrotaline	AAV1	Intratracheal	↓ RVSP ↓ mPAP ↓ RV hypertrophy ↓ Vascular remodeling	[105]
*ENOS*	Chronic hypoxia	Adenovirus	Intratracheal	↓ mPAP ↓ RV hypertrophy ↓ PVR	[107]
*KV1.5*	Chronic hypoxia	Adenovirus	Intratracheal	↑ Cardiac function ↓ PVR ↓ RV hypertrophy ↓ Vasoconstriction	[114]
*SURVIVIN*	Monocrotaline	Adenovirus	Intratracheal	↓Vascular remodeling ↓ PVR ↓ RV hypertrophy	[115]
*CGRP*	Chronic hypoxia	Adenovirus	Intratracheal	↓RV hypertrophy ↓Vascular remodeling ↓ PVR	[123,124]

Adeno-associated virus serotype 1(AAV1); ATPase sarcoplasmic/endoplasmic reticulum Ca^2+^ transporting 2 (Serca2a); bone morphogenetic protein receptor type 2 (Bmpr2); calcitonin gene-related peptide (Cgrp); nitric oxide synthase 3 (Enos); potassium voltage-gated channel subfamily A member 5 (Kv1.5); mean pulmonary arterial pressure (mPAP); pulmonary vascular resistance (PVR); right ventricle (RV); right ventricular systolic pressure (RVSP); SIN3 transcription regulator family member A (Sin3a); decrease (↓); increase (↑).
